# Attitudes towards interprofessional education and associated factors among faculty at the college of health sciences in a public university in Kenya: a cross-sectional study

**DOI:** 10.11604/pamj.2022.42.4.32732

**Published:** 2022-05-05

**Authors:** Rosemary Kawira Kithuci, Drusilla Makworo, Albanus Mutisya, Justus Simba, Patrick Mburugu

**Affiliations:** 1 School of Nursing, Jomo Kenyatta University of Agriculture and Technology (JKUAT), Juja, Kenya,; 2 School of Medicine, Jomo Kenyatta University of Agriculture and Technology (JKUAT), Juja, Kenya

**Keywords:** Interprofessional education, positive attitudes, faculty, curriculum

## Abstract

**Introduction:**

conforming health professional´s curricula and training to emerging needs and exponential growth in medical information and education is key. Interprofessional education is one such conformity. Faculty attitudes towards interprofessional education is a good predictor to their engagement. The study purpose is to determine attitudes of faculty and associated factors towards interprofessional education (IPE) at the College of Health Sciences of Jomo Kenyatta University of Agriculture and Technology.

**Methods:**

a cross-sectional study among 71 faculty was conducted. A 5-point Likert scale with three attitude subscales on IPE were used to collect data using stratified sampling method. Attitude was dichotomized with >75% as cut-off for positive attitude. Data was analyzed using SPSS version 25.0 software at 95% confidence level. Logistic regression was used to identify relationship between bio-demographic characteristics and attitude of faculty.

**Results:**

there were more male faculty than females and the mean age was 42 years. The overall attitude score was positive (124.46 >75%), with attitudes of faculty towards IPE in academic settings subscale yielding negative attitude score (36.86 <75%). Age, gender, academic position, and expertise level were not significant in influencing faculty´s attitude. Application of interprofessional education was significant (P=0.036), with faculty who had applied Interprofessional education at the college more likely to have positive attitudes.

**Conclusion:**

faculty have overall positive attitudes towards interprofessional education but negative attitudes towards subscale 3-interprofessional education in academic settings. Behavior change training and IPE sensitization to avert negative attitudes among faculty is recommended.

## Introduction

The demographic, epidemiology, socioeconomic and technological environment within which health care professional practice keep changing [1]. The curriculum implementation period has remained constant over the decades despite exponential growth in medical information [2]. While it may not be possible to change curricula with every change in the health system, delivering the curricula innovately in a manner that embraces these complexities would be helpful [3]. One innovation has been interprofessional education (IPE). IPE is an experience that occurs when students from two or more professions learn about, from and with each other to enable effective collaboration subsequently improving health outcomes [1].

A systemic review on IPE revealed it is occurring in several countries mostly developed nations like United States of America (USA), Australia, Canada, Sweden, United Kingdom (UK), Norway, Poland, Belgium and Malaysia. It is also happening in some developing countries like Ghana, Egypt, South Africa, Ethiopia, Algeria, Uganda and Namibia though there is limited literature on the same [4]. Studies in developed countries have reported positive attitudes towards IPE with emphasis for need of training faculty on IPE [5,6]. In the Middle East, studies in Iraq and United Arab Emirates (UAE) reported positive attitudes on IPE among faculty [7,8]. In Africa, IPE is not well established [9]. African Interprofessional Education Network (AfrIPEN) is putting concerted efforts towards IPE in Africa [9].

Interprofessional education among faculty models practice behavior to students while they still in training. When faculty embrace IPE, its benefits will trickle down to students they teach. Some benefits of IPE to students include teamwork, improves interpersonal relations, helps break professional boundaries and fosters collaborative management [10,11]. Despite these benefits, some students feel IPE is hectic and adds no value (negative training mindset), it is not a course requirement and that their professional programmes have tight schedules and timelines to accommodate IPE [12,13]. Where learning structures and resources (time, materials and money) are constrained as its mostly the case in developing countries, implementing IPE is harder [11,12,14]. Despite advocacy by bodies like World Health Organization and Institute of Medicine, interprofessional education hasn´t been widely adopted more so in low- and middle-income countries like Kenya. Professions continue to train uniprofessionally and remain encapsulated in their professional cocoons that have been otherwise hard to break. Stereotypes do exist among professions and these hinder interaction for maximum utilization of teams. In an attempt to integrate IPE into curricula, faculty´s attitudes can´t be ignored as it´s a good predictor to acceptability [8].

The purpose of this study is to determine faculty´s attitudes and the sociodemographic characteristics influencing attitude of faculty towards IPE at a Kenyan public university. The findings will inform policy in developing IPE content and modalities and inform training needs before commencing of an IPE program at Jomo Kenyatta University of Agriculture and Technology (JKUAT).

## Methods

**Study design:** this study adopted a cross-sectional design. Data was collected from faculty on attitudes towards IPE at one point in time.

**Setting:** this study was conducted at JKUAT, College of Health Sciences in Kiambu County, Kenya. The five schools, that is: School of Medicine, School of Biomedical Sciences, School of Pharmacy, School of Nursing and School of Public Health were included. The college trains a wide mix of health care professionals that share learning infrastructure and clinical learning sites. However, there is no structured IPE initiatives in the training curricula. The study was conducted between June and August 2020.

**Sample size and sampling technique:** a calculated sample size of 88 using Cochrane equation was used in this study [15]. Seventy-one (71) faculty from the five schools at the college of health sciences participated in the study translating to 81% response rate. Stratified sampling was used to select respondents where schools were strata and simple random sampling used to pick respondents in schools. Proportionate allocation of sample to schools was applied. A probability sampling method was preferred to control for bias.

### Eligibility criteria

**Inclusion:** full time faculty members in the college of health sciences.

**Exclusion:** faculty members heading the schools and college (deans and principal) and the researcher´s and academic supervisors.

**Study variables:** independent variable: bio-demographic characteristics of faculty. Dependent variable: attitude score.

**Study outcome:** this study determined faculty´s attitudes towards IPE as a score using modified Blooms cut off scores [16].

**Tools and procedures:** a 30 items 5-point attitude Likert scale with 3 subscales was adapted to the local setting and used in this study. The scales are: attitudes towards IPE scale, attitudes towards health care teams (ATHCT) scale and attitudes towards IPE in academic settings scale. The original tools are available at the National Centre for Interprofessional Practice and Education (Nexus) Resource Centre, have no copyright or license and permission to use them was sought. The tools were administered online to selected faculty using their official email. A reminder was sent in intervals of one week to those who had not responded to either fill or decline to participate in the study.

**Data management and analysis:** data cleaning was done prior to analysis that included exclusion of incomplete tools from analysis. Data was analyzed using SPSS version 25.0 for descriptive statistics. Statistical significance was considered at 95% confidence interval. Attitude minimum score was 30 with maximum score as 150. Scores were converted into percentages and ≥75% (≥112/150) overall or ≥38/50 per scale or ≥3.8/5 per statement was considered positive attitudes with <75% (30-112) overall score or 10-37 per scale or less than 3.8/5 considered negative attitudes according to modified Blooms cuff point [16]. Shift from centrality bias was considered when determining cutoff and negative statements were reverse scored during analysis. Data from age, years of experience in profession and years of experience in teaching which was obtained was continuous data was categorised for analysis. Binary logistic regression was used to determine any relationship between biodemographic characteristics of age, gender, years of experience in the profession, years of experience in teaching, academic position, school, expertise level of faculty and their attitude towards IPE among faculty. Chi-square test of association was used to analyse support towards IPE among faculty and attitude.

**Ethical considerations:** ethical clearance was sought from JKUAT Institutional Research Ethics Committee (IREC) and a research permit sought from National Commission of Science Technology and Innovation (NACOSTI). Permission was sought from JKUAT administration before commencement of data collection and confidentiality and anonymity of respondents was always maintained.

## Results

**Bio-demographic characteristics:** out of the 88 participants sampled, 72 participated in the study and 71 had complete questionnaires that were used in analysis. This translated to 81% response rate. Data collection was done at the peak of COVID-19 pandemic, when the university was closed, and this could have led to the inability to achieve 100% response rate.

Majority 41 (57.7%) of the participants were males. About half, 37 (52%) of the respondents were aged 35-44 years, 38 (53.5%) had between 10-19 years of experience in the profession and 64 (90.1%) had between 1-10 years of experience in teaching. The school of medicine had almost half 32 (45.1%) of the respondents, which was proportionate to the school size followed by nursing 13 (18.3), school of public health and biomedical sciences 10 (14.1%) each and the school of pharmacy had 6 (8.5%). More faculty, 33 (46.5%) were in the middle level lecturer academic position, 21 (29.6%) were assistant lecturers, 13 (18.3%) were senior lecturers, 2 (2.8%) were graduate assistants and 2 (2.8%) were associate professors. On IPE expertise, 42 (59.2%) were novices with little familiarity on IPE, 21 (29.6%) had no familiarity on IPE at all whiles, 8 (11.3%) were experienced. This is summarized in Table 1.

**Table 1:** bio-demographic characteristics of the respondents

Variables (n=71)		% (n) or mean (SD)
Gender	Female	42.3 (30)
	Male	57.7 (41)
Academic position	Graduate assistant	2.8 (2)
	Tutorial fellow/assistant lecturer	29.6 (21)
	Lecturer	46.5 (33)
	Senior lecturer	18.3 (13)
	Associate professor	2.8 (2)
School	Public health	14.1 (10)
	Medicine	45.1 (32)
	Nursing	18.3 (13)
	Biomedical sciences	14.1 (10)
	Pharmacy	8.5 (6)
Age in years	24-34 years	15.5 (11)
	35-44 years	52.1 (37)
	45-54 years	31 (22)
	55-65 years	1.4 (1)
Years of experience in health profession	0-9 years	12.9 (9)
	10-19 years	53.5 (38)
	20-29 years	40 (22)
	30-39 years	2.8 (2)
Years of experience in teaching	1-10 years	90.1 (64)
	11-20 years	8.45 (6)
	21-30 years	1.4 (1)
Expertise Level	Not familiar	21 (29.6)
	Novice (some familiarity)	42 (59.2)
	Experienced	8 (11.3)
Application of IPE at JKUAT	Yes	18 (25.4)
	No	48 (67.6)
	N/A	5 (7)
Support IPE	Yes	66 (93)
	No	5 (7)

More than half, 48 (67.6%) of the participants hadn´t applied IPE at JKUAT, 18 (25.4%) had applied while 5 (7) were not aware of IPE at all. Majority 66 (93%) of faculty would support IPE initiatives. They felt it would foster teamwork and collaborative practice, improve quality of training, and enable good resource utilization as schools have shared infrastructure. Five (7%) of faculty wouldn´t support IPE as the syllabus in the schools differed, it was time consuming, and their professional identity would be lost (Table 1).

**Attitudes of IPE among faculty:** the mean attitude score was 124.46 (SD 9.7, SE 1.55). Attitudes towards IPE scale yielded a mean score of 44.76 (>75%), IPE attitudes among healthcare teams (ATHCT) scale IPE score was 42.84 (>75%) and attitudes of IPE in academic setting scale score was 36.86 (<75%). Slightly more than half 37 (52.1%) of the respondents had positive attitude. While the overall attitude score and that of two subscales was positive, attitudes of IPE in academic setting subscale yielded a negative score. Items 12 (3.04), 22 (3.11), 23 (3.18), 25 (2.87), 26 (3.06), 29 (3.54) and 30 (3.59) yielded negative scores (Table 2).

**Table 2:** attitude towards interprofessional education subscales

No	Sub scale 1: attitudes towards interprofessional education	N	Min	Max	Mean	Std error	Std. dev
1	Interprofessional learning will help students think positively about other health professionals	71	3	5	4.65	0.07	0.56
2	Students in my professional group would benefit from working on small-group projects with other health profession students	71	3	5	4.56	0.07	0.58
3	Communications skills should be learned with integrated classes of health care students	71	3	5	4.62	0.06	0.54
4	Interprofessional learning will help to clarify the nature of patient problems for students	71	2	5	4.37	0.09	0.74
5	It is not necessary for undergraduate health care students to learn together	71	1	5	4.21	0.12	0.97
6	Learning with students in other health professional schools helps learners to become more effective members of a health care team	71	1	5	4.49	0.09	0.79
7	Interprofessional learning among health care students will increase their ability to understand clinical problems	71	2	5	4.39	0.086	0.73
8	Interprofessional learning will help students to understand their own professional limitations	71	2	5	4.30	0.09	0.76
9	Interprofessional learning among health professional students will help them to communicate better with patients and other professionals	71	3	5	4.47	0.07	0.56
10	Team-working skills are essential for all health care students to learn	71	3	5	4.7	0.06	0.49
	Totals		23	50	44.76		
**No**.	**Sub scale 2: attitudes towards interprofessional health care teams items**	**N**	**Min**	**Max**	**Mean**	**Std error**	**Std. dev**
1	Clients receiving Interprofessional care are more likely than others to be treated as whole persons	71	3	5	4.58	0.07	0.62
2	Developing an interprofessional client care plan is time-consuming	71	1	5	3.04	0.12	1.03
3	Interprofessional approach makes the delivery of care more efficient	71	2	5	4.48	0.08	0.65
4	Developing a client care plan with other team members avoids errors in delivering care improving decision making	71	2	5	4.48	0.08	0.65
5	Working in an interprofessional manner unnecessarily complicates things most times	71	2	5	4.14	0.10	0.82
6	The interprofessional approach improves the quality of care to clients	71	3	5	4.5	0.07	0.60
7	Health professionals working as teams are more responsive than others to the emotional and financial needs of clients	71	2	5	4.06	0.11	0.94
8	Having to report observations to a team helps team members better understand the work of other health professionals	71	2	5	4.5	0.08	0.65
9	Hospital patients who receive interprofessional team care are better prepared for discharge than other patients	71	2	5	4.48	0.09	0.73
10	Team meetings foster communication among members from different professions or disciplines	71	3	5	4.58	0.07	0.58
	Totals		22	50	42.84		
**No**	**Sub scale 3: attitudes towards interprofessional learning in the academic setting**	**N**	**Min**	**Max**	**Mean**	**Std error**	**Std. dev**
1	Interprofessional learning better utilizes resources	71	3	5	4.51	0.07	0.56
2	Students like courses taught by faculty from other academic departments	71	1	5	3.11	0.11	0.99
3	Students like courses that include students from other academic departments	71	1	5	3.18	0.10	0.83
4	Faculty at COHES should be urged to participate in interprofessional courses	71	2	5	4.24	0.08	0.71
5	Faculty like teaching students in other academic departments	71	1	4	2.87	0.09	0.74
6	Faculty like teaching with faculty from other academic departments	71	1	5	3.06	0.09	0.77
7	Interprofessional efforts weaken course content	71	1	5	4.169	0.08	0.70
8	Interprofessional efforts require support from college/university administration	71	2	5	4.58	0.071	0.60
9	Faculty should be rewarded for participation in interprofessional courses	71	2	5	3.54	0.09	0.82
10	Accreditation requirements limit interprofessional efforts	71	1	5	3.59	0.13	1.06
			15	49	36.86		
	Total attitude score	71	92	139	124.46	1.55	9.7

Scale 1 and 2 reported positive attitudes while scale 3 recorded negative attitudes of faculty towards IPE; the overall attitude (average of the three scales) was positive; COHES: college of health sciences

Further, using binary logistic regression, the respondent´s gender, age, years of experience in the profession, years of experience in teaching, school of affiliation, academic position did not significantly influence their attitude towards IPE. Though not significant (p=0.061), faculty´s classified as novices in IPE on expertise level were 5.3 times more likely to have positive IPE attitude than those who were not familiar with IPE (OR 5.3; 95% CI 0.923-30.644). There was a statistically significant association between applying IPE and faculty´s attitude (P=0.036). Faculty who applied IPE at the college of health sciences were 3.8 times more likely to have positive attitudes towards IPE than those who didn´t (OR 3.8; 95% CI 1.093-13.24) (Table 3).

**Table 3:** relationship between demographic characteristics and attitude towards IPE among faculty

Variables		Attitude		B	Sig.	COR	95% C.I.for EXP (B)	
		+ve	-ve				Lower	Upper
Gender	Female	17	13	-0.317	0.512	0.728	0.283	1.877
	Male	20	21	Ref				
Age group	25 - 34	6	5	-21.385	1	0	0	.
	35 - 44	23	14	-21.699	1	0	0	.
	45 - 54	8	14	-20.643	1	0	0	.
	55 - 64	0	1	Ref				
Academic position	Graduate assistant	2	0	-21.203	0.998			
	TF/assistant lecturer	11	10	-0.095	0.999	0	0	.
	Lecturer	16	17	0.061	0.949	0.909	0.05	16.54
	Senior lecturer	7	6	-0.154	0.967	1.063	0.061	18.454
	Associate professor	1	1	Ref.				
School of affiliation	Public health	5	5	0	1	1	0.132	7.57
	Medicine	19	13	-0.379	0.671	0.684	0.119	3.933
	Nursing	7	6	-0.154	0.876	0.857	0.124	5.944
	BioMed sciences	3	7	0.847	0.428	2.333	0.287	18.965
	Pharmacy	3	3	Ref				
Years as health profession	10-19	24	14	0.693	0.661	2	0.90	44.35
	20-29	9	13	-0.539	0.711	0.583	0.34	10.07
	30-39	1	1	0.369	0.804	1.44	0.8	26.23
	0-9	3	6	Ref				
Years in teaching	11-20	3	3	21.14	1	1.5x10^9^	60	-
	21-30	1	0	21.203	1	1.6x10^9^	00	-
	1-10	33	31	Ref				
Current expertise in IPE	Novice	27	15	1.674	0.061	5.333	0.928	30.644
	Experienced	5	3	-0.077	0.923	0.926	0.194	4.425
	Not familiar	5	16	Ref				
Application of IPE	No	23	25	22.456	0.999	5.65x10^9^	0	.
	Yes	14	4	1.336	0.036	3.804	1.093	13.241
	N/A	0	5	Ref				

TF: teaching fellow; N/A: not applicable

There was a statistically significant association between supporting students from different profession and attitude (P=0.021). Respondents who supported different professions learning together were 2.3 times more likely to have a positive attitude as compared to those who didn´t support (OR 2.3; 95% CI 1.733 to 2.989) (Table 4).

**Table 4:** relationship between supporting students from different profession learning together and attitude (cross-tabulation)

		Attitude		Total	Chi-square value	df	OR	CI	p-value
		Positive	Negative		Fischer's exact test				
Supporting students from different profession learning together	No	0	5	5	5.83	1	2.3		0.021
	Yes	37	29	66					
Total		37	34	71					

## Discussion

There was no statistically significant difference between the bio-demographic characteristics of gender, age, years of experience as health professional and years of experience as educators, academic position and school of affiliation and attitude towards IPE. On faculty´s expertise level (P=0.061), novices were 5.3 times more likely to have positive attitudes than those who were not familiar to IPE in this study. In a UAE study, being female faculty and having prior experience in IPE were significant in influencing IPE, while age, years of experience as a health professional educator influenced attitudes towards IPE though not to statistical significance with those between 30 to 50 years and having more than 5 years´ experience having higher scores [7]. In a Saudi Arabia study, being female and aged 41-50 years significantly influenced faculty´s attitude towards IPE [6]. Years of experience was not significant in this study which is contrary to a study in two universities in rural United States of America that showed negative correlation between years of experience and attitude with those who have more years of experience having negatives attitudes explained maybe by the historical uniprofessional training of health professionals [17].

In yet another study years of experience did not influence faculty´s attitude [18]. Faculty older in the profession are stuck in the uniprofessional way of training they were trained with, and this would explain why years of experience and age had no influence on attitude. There is no structured IPE in JKUAT, no school implement it giving a reason why school of affiliation was not significant. School of affiliation was significant in influencing IPE in an UAE study with the school of nursing reporting higher scores [7]. Students from the school of nursing and school of medicine work closely in the clinical areas and in practice compared to other schools in this study and this has made early studies to be done in these schools in most settings. This could explain why these two schools showed higher scores. The faculty who were experienced and novices had undergone training on IPE or participated in IPE related grants at the college and hence had better attitudes.

Faculty who had applied IPE at JKUAT had better attitudes than those who hadn´t (p=0.036). This points out to a link between awareness and knowledge of IPE and attitude. Further, faculty who supported IPE were 2.3 times more likely to have positive attitudes compared to those who didn´t. (P=0.021). This is a key predictor of acceptability before commencing of IPE initiatives and integration into curricula. IPE fosters interprofessional relationships and therefore, there is need to put structures that would foster IPE to harness on the positive attitudes from faculty [17]. Inculcating a culture of IPE among faculty would help harness IPE core competences of value and ethics, roles and responsibilities, communication and teamwork [19].

The mean overall attitude score was 124.46 >75%. This denotes faculty from this study had positive attitudes towards IPE. Attitude to a large extent shapes acceptance and adoptability of ideas and in this case IPE. Several studies in Iraq, USA, Korea, UAE have reported positive attitudes towards IPE [4-7,10-12,20]. However, sub scale 3 on attitudes of IPE in academic setting scale yielded a negative score (36.86 <75%). Salama (2018) in their study reported lower scores in this subscale though not negative scores as observed in the current study [7]. This brings out the fact that while faculty would embrace IPE, they weren´t sure of its suitability and adoptability in academic settings especially on teaching students and with faculty from other schools further pointing out to professional stereotypes and cocoons that still do exist. Efforts need to be put to avert these negative sentiments before adoption of IPE into training.

There is no structured IPE programme at JKUAT and therefore faculty responses were based on their knowledge and attitudes acquired elsewhere. Future research on the same is recommended upon implementation of an IPE program to compare attitude scores.

**Limitations:** this study is conducted in one public university and therefore can’t be generalized to other universities in Kenya. The findings however, are useful in informing the status of attitudes towards IPE and can be inferred in similar settings. The study was done at the peak of the COVID-19 pandemic and that may have caused inability to achieve full sample. The achieved response rate is however, acceptable for descriptive research [21].

## Conclusion

Overall attitudes towards IPE among faculty was positive however, attitudes towards IPE in academic settings was negative. Age, gender, academic position, school of affiliation and years of experience as faculty and in teaching did not influence faculty´s attitude towards IPE. Faculty´s expertise level and having applied IPE at the college influenced their attitudes towards IPE. Behavior change training and sensitization on IPE among faculty is recommended.

**Funding:** this study received no direct funding, however, the correspondent author received tuition waiver for PhD academic work from JKUAT.

### What is known about this topic

- Attitudes towards IPE has been widely studied in other parts of the world with some studies using same tools as used in this study;
- Factors associated with attitude towards IPE have also been studied in other studies.

### What this study adds

- Despite many studies of IPE elsewhere, none exist in Kenya and there being no structured IPE at JKUAT this study brings in insights into the area and what can be done to improve on attitudes during adoption of IPE into curricula;
- The associated factors in our setup are not known.
